# Supplementary material for: Ancient origin of the rod bipolar cell pathway in the vertebrate retina
Source: bioRxiv. 2023 Oct 4:2023.09.12.557433. Originally published 2023 Sep 13. Preprint. [Version 2] doi: 10.1101/2023.09.12.557433 (PMC10525478; doi:10.1101/2023.09.12.557433)
Supplement: Supplement 1 [file NIHPP2023.09.12.557433v2-supplement-1.pdf]

bioRxiv preprint doi: https://doi.org/10.1101/2023.09.12.557433; this version posted October 4, 2023. The copyright holder for this preprint (which was not certified by peer review) is the author/funder, who has granted bioRxiv a license to display the preprint in perpetuity. It is made available under aCC-BY 4.0 International license.

Figure S1

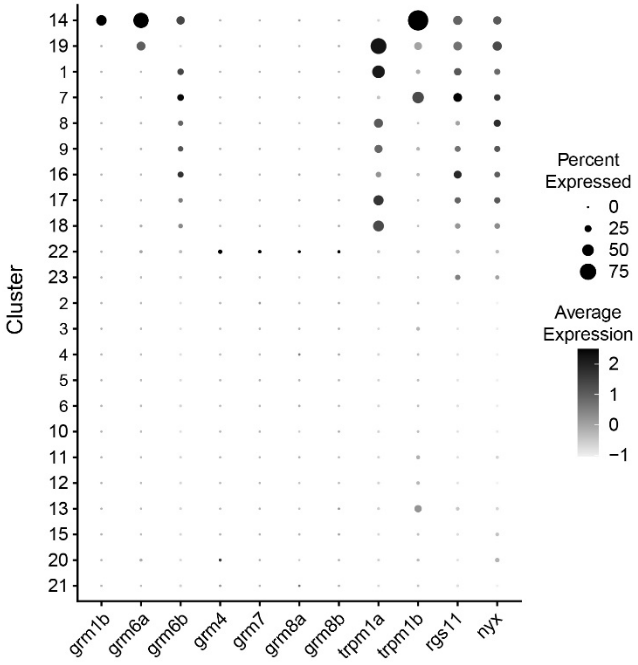

Figure S1. Expression patterns of the identified marker genes in BC clusters

The size of each circle depicts the percentage of cells in the cluster in which the marker was detected (≥1 UMI), and its grey scale depicts the scaled average expression level of cells within the cluster

bioRxiv preprint doi: https://doi.org/10.1101/2023.09.12.557433; this version posted October 4, 2023. The copyright holder for this preprint (which was not certified by peer review) is the author/funder, who has granted bioRxiv a license to display the preprint in perpetuity. It is made available under aCC-BY 4.0 International license.

Figure S2

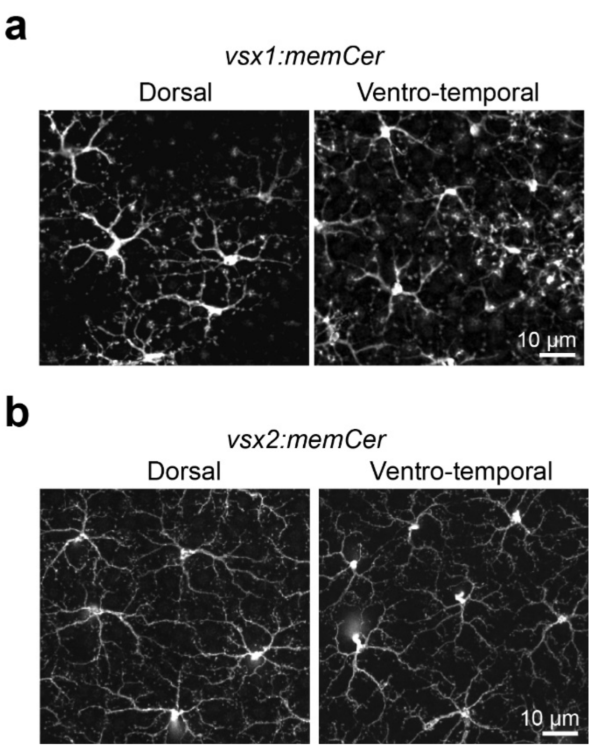

Figure S2. Dendritic tiling of RBC1 and RBC2 BCs across the retina

Confocal images of retinal flat mount at outer plexiform layer level from Tg(vsx1:memCerulean)^{q19} (vsx1:memCer) and Tg(vsx2:memCerulean)^{wst01} (vsx2:memCer). Note that the vsx1:memCer line occasionally labels OFF BCs. These BCs were distinguished by tracing the cells to the axon terminals in the confocal image volumes.

bioRxiv preprint doi: https://doi.org/10.1101/2023.09.12.557433; this version posted October 4, 2023. The copyright holder for this preprint (which was not certified by peer review) is the author/funder, who has granted bioRxiv a license to display the preprint in perpetuity. It is made available under aCC-BY 4.0 International license.

Figure S3

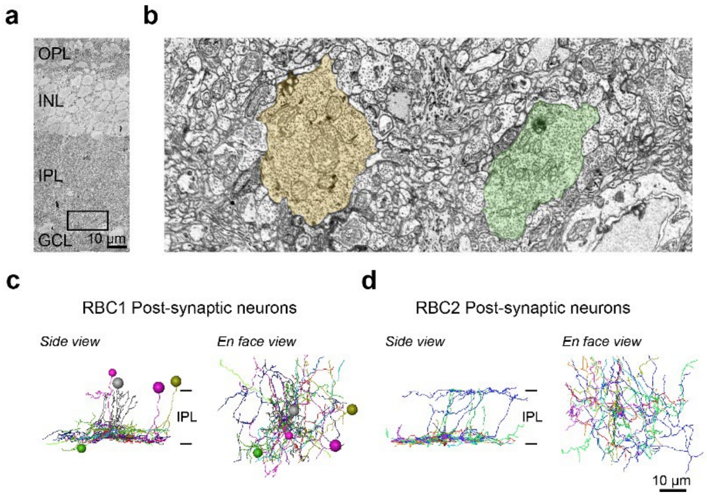

Figure S3. Identification of RBC1 and RBC2 postsynaptic neurons in SBFSEM volume

***a**, A partial image of an example SEM image of an adult zebrafish retina. OPL (outer plexiform layer), INL (inner nuclear layer), IPL (inner plexiform layer), GCL (ganglion cell*

bioRxiv preprint doi: https://doi.org/10.1101/2023.09.12.557433; this version posted October 4, 2023. The copyright holder for this preprint (which was not certified by peer review) is the author/funder, who has granted bioRxiv a license to display the preprint in perpetuity. It is made available under aCC-BY 4.0 International license.

layer). ***b****, Magnified image of the region within the black box in* ***a*** *at the bottom layer of the IPL. Characteristic large bipolar cell axons are painted in light yellow and green.* ***c,d,*** *Traces of neuronal processes and the location of somas of cells that are post-synaptic to RBC1 and RBC2 cells. Individual cells were color coded. IPL: inner plexiform layer.*

bioRxiv preprint doi: https://doi.org/10.1101/2023.09.12.557433; this version posted October 4, 2023. The copyright holder for this preprint (which was not certified by peer review) is the author/funder, who has granted bioRxiv a license to display the preprint in perpetuity. It is made available under aCC-BY 4.0 International license.

Figure S4

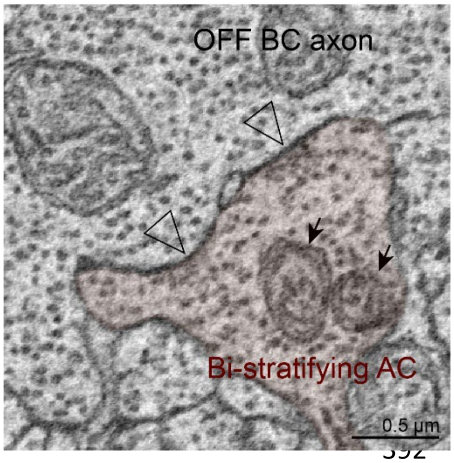

Figure S4. Ultrastructure of the zebrafish A2 AC boutons in the OFF layer

Examples of large synaptic sites (arrowheads) between bi-stratifying A2-like AC and OFF BC axon terminals in the OFF layer. The A2 AC boutons often contain mitochondria (arrows).

bioRxiv preprint doi: https://doi.org/10.1101/2023.09.12.557433; this version posted October 4, 2023. The copyright holder for this preprint (which was not certified by peer review) is the author/funder, who has granted bioRxiv a license to display the preprint in perpetuity. It is made available under aCC-BY 4.0 International license.

Figure S5

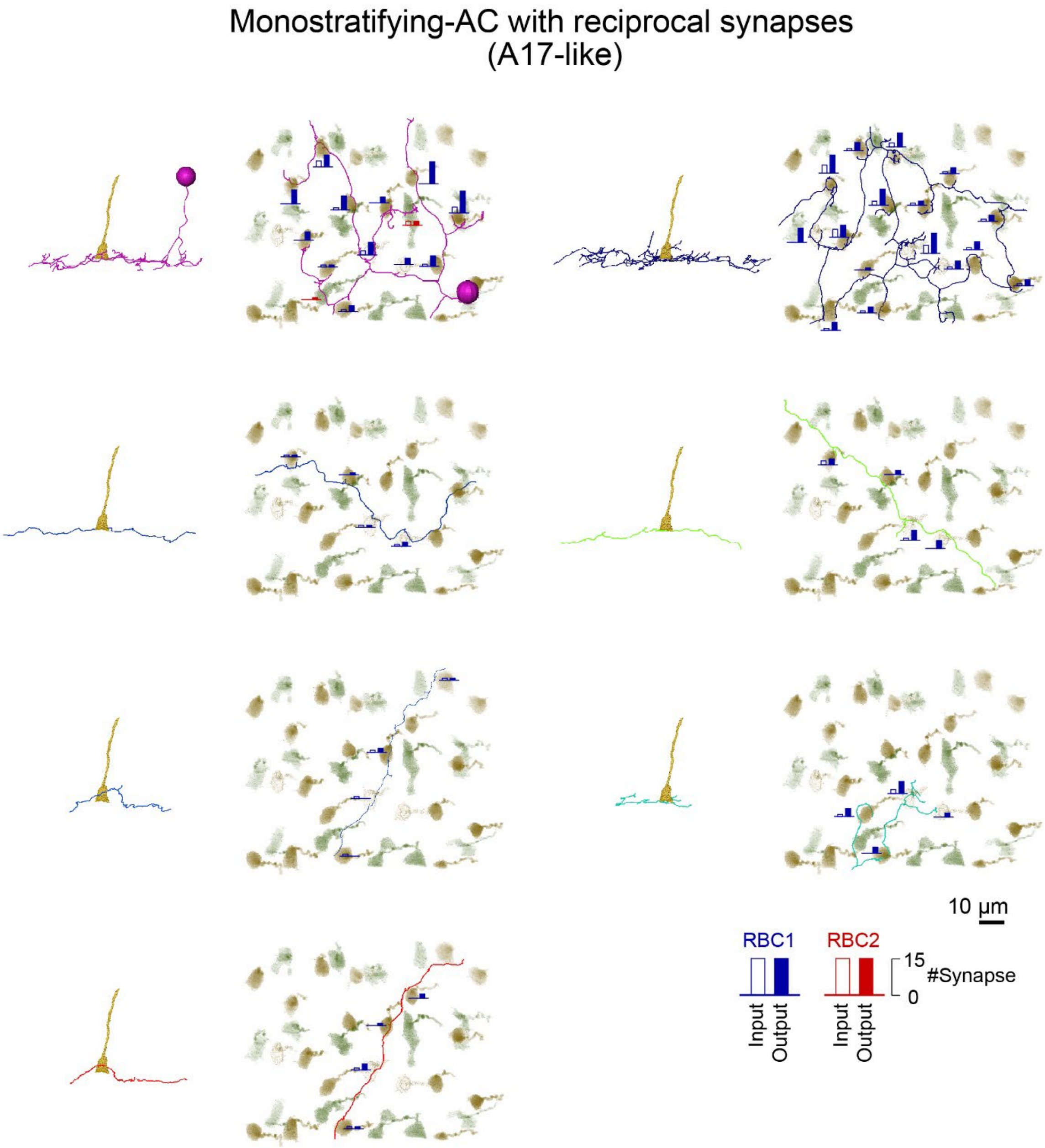

752

753 **Figure S5. Gallery of mono-stratifying AC making reciprocal synapses with RBC1**

754 *En face* and side views of individual cells or processes. The numbers of input (open bar) and
755 output (closed bar) synapses with each RBC1 (blue) and RBC2 (red) terminal are indicated as
756 the height of the bars.

bioRxiv preprint doi: https://doi.org/10.1101/2023.09.12.557433; this version posted October 4, 2023. The copyright holder for this preprint (which was not certified by peer review) is the author/funder, who has granted bioRxiv a license to display the preprint in perpetuity. It is made available under aCC-BY 4.0 International license.

Figure S6

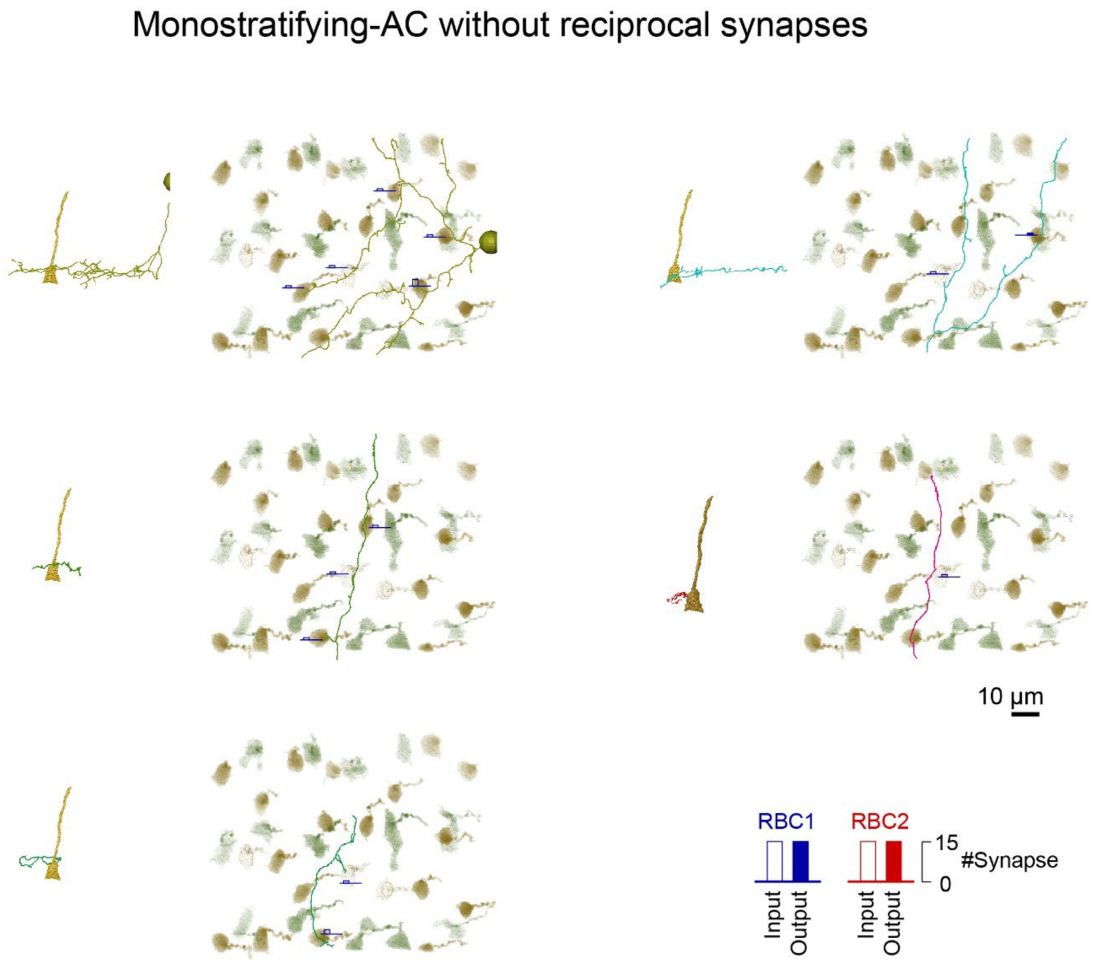

757

758 **Figure S6. Gallery of mono-stratifying AC without reciprocal synapses with RBC1**

759 *En face* and side views of individual cells. The numbers of input (open bar) and output (closed
760 bar) synapses with each RBC1 (blue) and RBC2 (red) terminal are indicated as the height of the
761 bars.

762

763

764

bioRxiv preprint doi: https://doi.org/10.1101/2023.09.12.557433; this version posted October 4, 2023. The copyright holder for this preprint (which was not certified by peer review) is the author/funder, who has granted bioRxiv a license to display the preprint in perpetuity. It is made available under aCC-BY 4.0 International license.

Figure S7

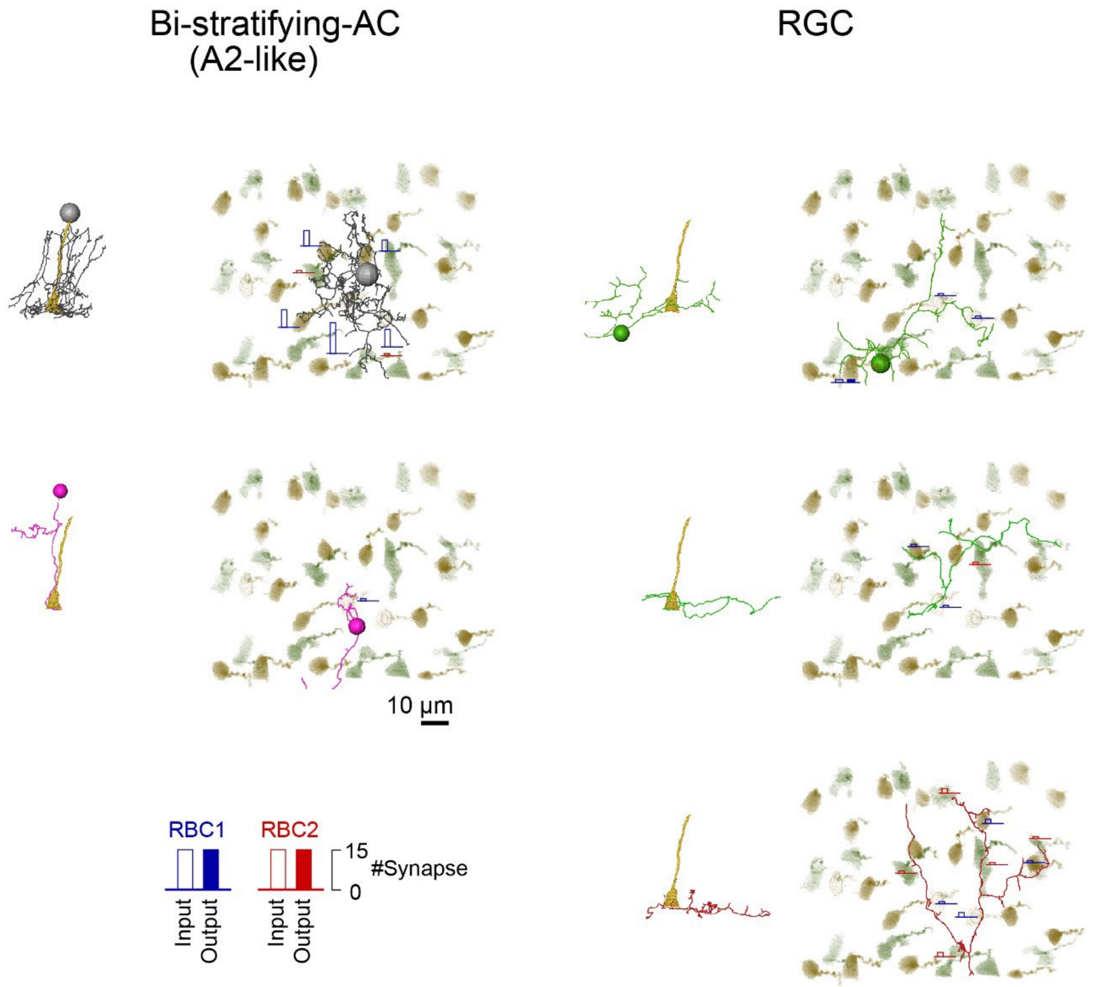

Figure S7. Gallery of bi-stratifying AC and RGC contacted to RBC1

En face and side views of individual cells. The numbers of input (open bar) and output (closed bar) synapses with each RBC1 (blue) and RBC2 (red) terminal are indicated as the height of the bars.

bioRxiv preprint doi: https://doi.org/10.1101/2023.09.12.557433; this version posted October 4, 2023. The copyright holder for this preprint (which was not certified by peer review) is the author/funder, who has granted bioRxiv a license to display the preprint in perpetuity. It is made available under aCC-BY 4.0 International license.

Figure S8

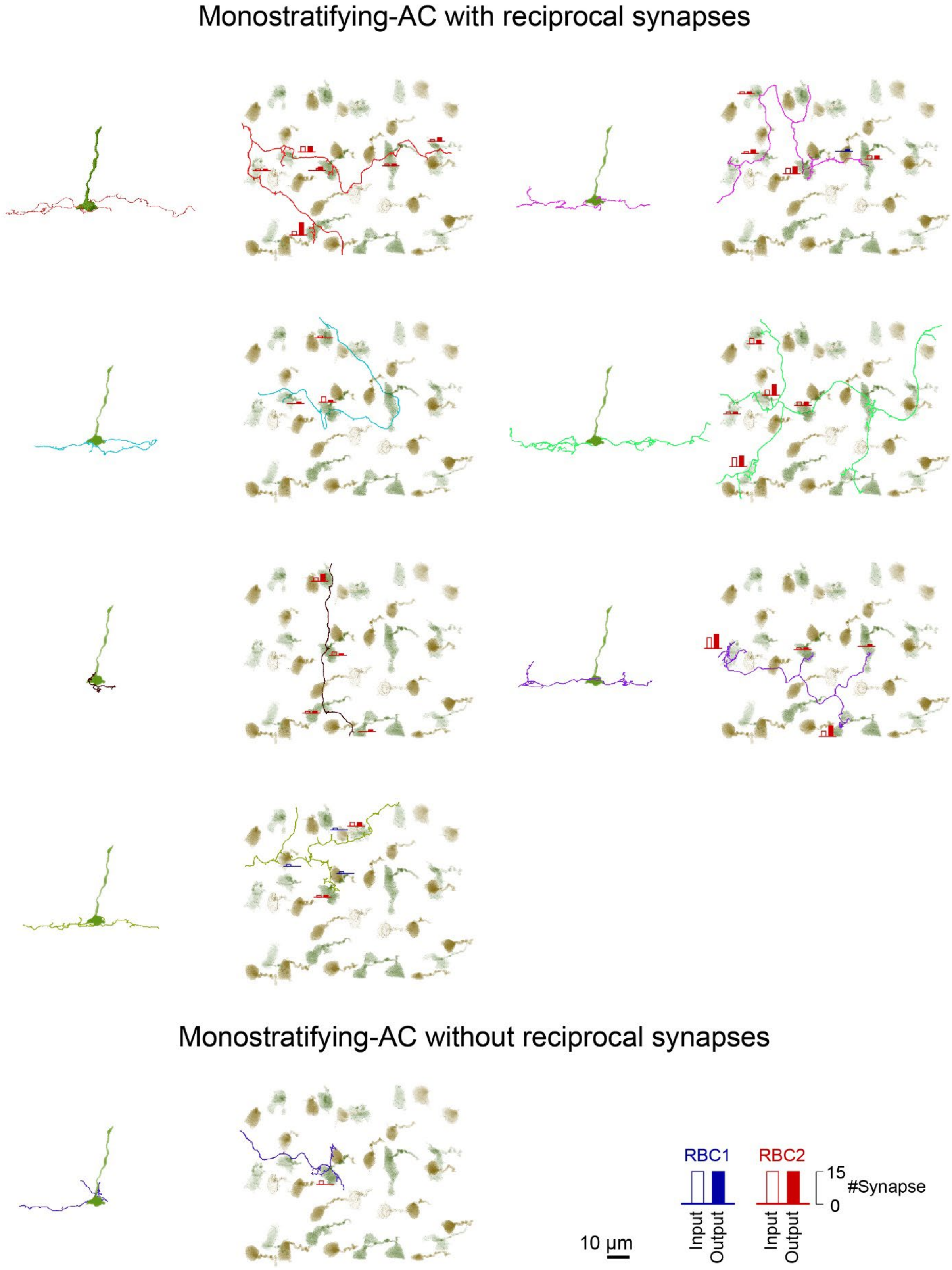

773

bioRxiv preprint doi: https://doi.org/10.1101/2023.09.12.557433; this version posted October 4, 2023. The copyright holder for this preprint (which was not certified by peer review) is the author/funder, who has granted bioRxiv a license to display the preprint in perpetuity. It is made available under aCC-BY 4.0 International license.

Figure S8. Gallery of mono-stratifying AC connected to RBC2

En face and side views of individual cells. The numbers of input (open bar) and output (closed bar) synapses with each RBC1 (blue) and RBC2 (red) terminal are indicated as the height of the bars.

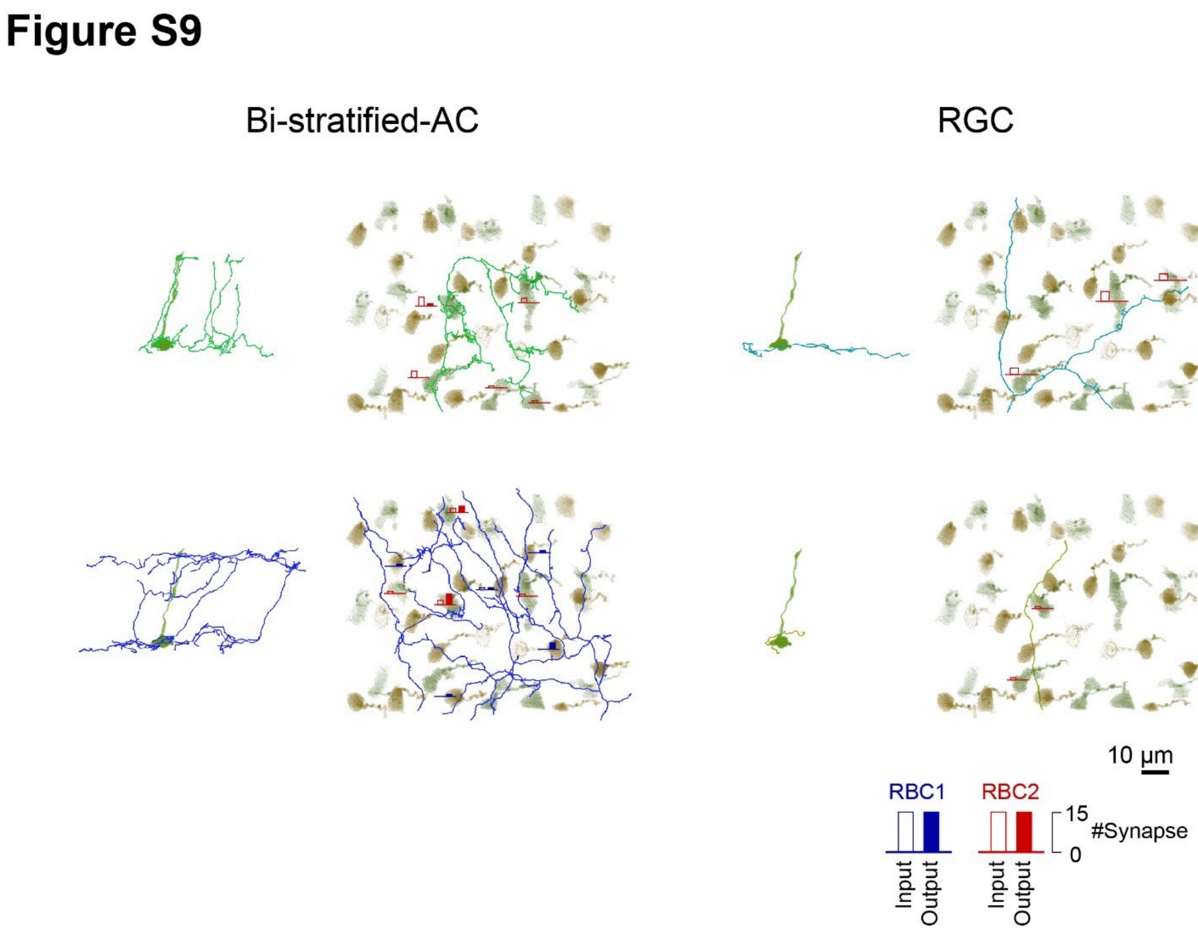

Figure S9. Gallery of bi-stratifying AC and RGC connected to RBC2

En face and side views of individual cells. The numbers of input (open bar) and output (closed bar) synapses with each RBC1 (blue) and RBC2 (red) terminal are indicated as the height of the bars.
